# Evaluation of *in vitro* culture systems for the maintenance of microfilariae and infective larvae of *Loa loa*

**DOI:** 10.1186/s13071-018-2852-2

**Published:** 2018-05-02

**Authors:** Denis Zofou, Fanny Fri Fombad, Narcisse V. T. Gandjui, Abdel Jelil Njouendou, Arnaud Jonas Kengne-Ouafo, Patrick W. Chounna Ndongmo, Fabrice R. Datchoua-Poutcheu, Peter A. Enyong, Dizzle Tayong Bita, Mark J. Taylor, Joseph D. Turner, Samuel Wanji

**Affiliations:** 1 Research Foundation for Tropical Diseases and Environment (REFOTDE), South West Region, Buea, Cameroon; 2 Biotechnology Unit, Faculty of Science, University of Buea, Buea, Cameroon; 3 Parasites and Vectors Biology Research Unit (PAVBRU), Department of Microbiology and Parasitology, Faculty of Science, University of Buea, South West Region, Buea, Cameroon; 4 Department of Parasitology, Liverpool School of Tropical Medicine, Liverpool, UK

**Keywords:** *Loa loa*, L3 larvae, Microfilariae, *In vitro* culture system, Viability, Moulting

## Abstract

**Background:**

Suitable and scalable *in vitro* culture conditions for parasite maintenance are needed to foster drug research for loiasis, one of the neglected tropical diseases which has attracted only limited attention over recent years, despite having important public health impacts. The present work aims to develop adequate *in vitro* culture systems for drug screening against both microfilariae (mf) and infective third-stage larvae (L3) of *Loa loa*.

**Methods:**

*In vitro* culture conditions were evaluated by varying three basic culture media: Roswell Park Memorial Institute (RPMI-1640), Dulbecco’s modified Eagle’s medium (DMEM) and Iscove’s modified Dulbecco’s medium (IMDM); four sera/proteins: newborn calf serum (NCS), foetal bovine serum (FBS), bovine serum albumin (BSA) and the lipid-enriched BSA (AlbuMax® II, ALB); and co-culture with the Monkey Kidney Epithelial Cell line (LLC-MK2) as a feeder layer. The various culture systems were tested on both mf and L3, using survival (% motile), motility (T_90_ = mean duration (days) at which at least 90% of parasites were fully active) and moulting rates of L3 as the major criteria. The general linear model regression analysis was performed to assess the contribution of each variable on the viability of *Loa loa* L3 and microfilarie. All statistical tests were performed at 95% confidence interval.

**Results:**

Of the three different media tested, DMEM and IMDM were the most suitable sustaining the maintenance of both *L. loa* L3 and mf. IMDM alone could sustain L3 for more than 5 days (T_90_ = 6.5 ± 1.1 day). Serum supplements and LLC-MK2 co-cultures significantly improved the survival of parasites in DMEM and IMDM. In co-cultures with LLC-MK2 cells, *L. loa* mf were maintained in each of the three basic media (T_90_ of 16.4–19.5 days) without any serum supplement. The most effective culture systems promoting significant moulting rate of L3 into L4 (at least 25%) with substantial maintenance time were: DMEM + BSA, DMEM + NCS, DMEM-AlbuMax®II, DMEM + FBS all in co-culture with LLC-MK2, and IMDM + BSA (1.5%), DMEM + FBS (10%) and DMEM + NCS (5%) without feeder cells. DMEM + 1% BSA in co-culture scored the highest moulting rate of 57 of 81 (70.37%). The factors that promoted *L. loa* mf viability included feeder cells (*β* = 0.490), both IMDM (*β* = 0.256) and DMEM (β = 0.198) media and the protein supplements NCS (*β* = 0.052) and FBS (*β* = 0.022); while for *L. loa* L3, in addition to feeder cells (*β* = 0.259) and both IMDM (*β* = 0.401) and DMEM (*β* = 0.385) media, the protein supplements BSA (*β* = 0.029) were found important in maintaining the worm motility.

**Conclusions:**

The findings from this work display a range of culture requirements for the maintenance of *Loa loa* stages, which are suitable for developing an effective platform for drug screening.

**Electronic supplementary material:**

The online version of this article (10.1186/s13071-018-2852-2) contains supplementary material, which is available to authorized users.

## Background

Loiasis is a parasitic disease caused by the filarial nematode *Loa loa* that is transmitted through the bite of an infected *Chrysops* fly. Loiasis is endemic in the rainforest areas of West and Central Africa [1]. The common clinical signs of loiasis are the subconjunctival migration of the adult worm, reported for the first time by Mongin in 1770 [2], Calabar Swelling, pruritis, oedemas and arthralgia. Interest in this filarial species, which has long been considered to be less pathogenic than related species [3], came from several reports in Cameroon indicating that high microfilaraemia of *L. loa* is associated with severe and sometimes fatal encephalopathic reactions in patients who had taken ivermectin for onchocerciasis treatment [4–7]. Loiasis is a neglected tropical disease (NTD) which has attracted only limited attention in drug research and development. Apart from surgical removal of adult worms moving under the skin or across the eye that can be done to relieve anxiety, only two medications have so far been employed for clinical cases since the last century, namely diethylcarbamazine (DEC) and albendazole. The latter is sometimes used in patients who are not cured with multiple DEC treatments. Several cases of brain inflammation, coma and death have been reported in people with heavy infections when they are treated with DEC [8, 9]. The risk of side effects has limited the deployment of mass drug administration of ivermectin in areas where the *L. loa* prevalence exceeds 20% [10–12], impeding the goals stated by the African Programme for Onchocerciasis Control (APOC) in areas of co-endemicity. Progress in drug research and development for loiasis requires suitable screening systems both at *in vitro* and *in vivo* levels. Though innovations in filarial animal models have recently been achieved [13, 14], *in vitro* maintenance systems of the different stages of *L. loa* have not been established. The present study aimed to design suitable *in vitro* culture systems for drug screening against both infective larvae (L3) and microfilariae (mf) of *L. loa*.

## Methods

### Isolation and purification of *L. loa* L3

*Loa loa* L3 were obtained from dissected *Chrysops* flies that had previously fed on a consented microfilaremic individual at Ediki Forest (South West region, Cameroon). Engorged *Chrysops* were kept in captivity for 12 days, to allow development to the infective stage (L3). The flies were fed daily with 15% sucrose solution soaked in cotton wool. After 12 days of rearing, the flies were dissected in Petri dishes containing RPMI 1640 medium (Sigma-Aldrich, St Louis, USA). The head, thorax and abdomen were separated and teased apart in three different Petri dishes. Fly tissues were incubated for 20 min to allow L3 larvae to migrate out. A sterile pipette was used to pick the larvae and pooled in a shallow convex glass dish [15]. The worms were transferred into 15 ml centrifuge tubes (Corning, Kennebunk-ME, USA) for purification. Only L3 harvested from the head (where more mature larvae are expected to be found) were used in this study. The remaining larvae were frozen to be used in other studies (immunology and molecular biology). The L3 were washed using a Percoll® (GE Healthcare, Pharmacia, Uppsala, Sweden) technique. The L3 suspension concentrated in less than 1 ml RPMI was slowly layered on the surface of a 15 ml tube containing stock iso-osmotic Percoll® and centrifuged (Humax 14k human, Germany) at 800× *rpm* for 10 min. The process was repeated to remove microbial contaminants. At the end, the L3 were washed twice with RPMI-1640 by centrifugation at 1500× *rpm* for 10 min to remove Percoll® remnant.

### Isolation and purification of *L. loa* mf

*Loa loa* mf were obtained from baboons (*Papio anubis*) experimentally infected with human strain of *L. loa* reared in Kumba Medical Research Station (South West region, Cameroon). Peripheral blood samples of hypermicrofilaraemic baboons were collected as described in the previous reports [13]. Microfilaraemic loads were determined microscopically on thick films. Calibrated thick blood smears were prepared by spreading a 50 μl venous blood sample from a 75 μl non-heparinised capillary tube, onto a clean slide over an area of 1.5 × 2.5 cm [16]. After drying, films were dehemoglobinized and stained with Giemsa. The Percoll® density centrifugation method previously described [17] was used to purify mf from infected blood samples.

### *In vitro* culture of parasites

Four supplements were used at 3 concentrations each: fetal bovine serum (Lonza) and newborn calf serum (Sigma-Aldrich, Berlin, Germany) at 15%, 10% and 5%; bovine serum albumin (Sigma-Aldrich, Berlin, Germany) and AlbuMax® II (Gibco Life Technologies, Cergy-Pontoise, France) at 1.5%, 1% and 0.5%. Three basic media were used: RPMI-1640 and IMDM (Sigma-Aldrich, St Louis, USA) and DMEM (Gibco Life Technologies, Cergy-Pontoise, France). Ciprofloxacin (5 μg/ml) was used as antibiotic and fluconazole (10 μg/ml) as antifungal. Flat bottom culture plates (48-well) with lids (Corning, Kennebunk, ME, USA) were loaded as follows: 800 μl of the different media with a range of 20–30 microfilariae or 10–15 larvae per well. Cultures were carried out in triplicates.

### Monkey kidney cell co-culture

Monkey kidney epithelial cells (LLC-MK_2_) (ATCC, USA) were cultured in flasks at 37 °C in a CO_2_ incubator (Sheldon Mfg. Inch, Cornelius, OR, USA) at 5% CO_2_ until the cell layer became fully confluent. For new inoculations and other cell manipulations, trypsin was used to detach cells from the walls of the flasks. Cells were then dislodged with trypsin solution (25%) containing EDTA, the mixture was kept at 37 °C for less than 1 h. The cell suspension was centrifuged at 1,500 rpm for 10 min, the supernatant was discarded, and the pellet re-suspended and diluted to 10^5^ cells/ml in complete culture medium. Aliquots (100 μl) of cell suspensions were plated into a 48-well culture plate and kept in the incubator for cells to become fully confluent.

### Assessment of parasite viability

The viability of the parasites was assessed daily, by visual inspection (by two individuals) under an inverted microscope until they die. Their motility was scored on a 4-point scale [18, 19]: 0, no movement or immotile; 1, intermittent shaking of head and tail; 2, sluggish (shaking of the whole worm on a spot); 3, vigorous movement (shaking of the whole worm and migration from one spot to was considered).

### Data processing and analysis

Three different batches of L3 larvae and microfilariae were used for each culture system. For each batch of parasites, 4 replicate wells were used per system. Raw data collected daily on record sheets were entered into a template designed on Microsoft Excel 2007. Three variables were defined and computed to assess the viability of the parasites (mean motility and mean mortality, T_90_).

Motility variable was computed based on the scoring system described above, and using the following formula.

$$ \mathrm{Motility}\ \left(\%\right)=\frac{\sum \mathrm{SiNi}}{3.\sum \mathrm{Ni}}\times 100 $$

where Si is the score of point scale i and Ni is the total number of worms at a point scale i.

The variable T_90_ was defined as the duration at which 90% of the worms were still fully active (score 3 above) in the well. This variable was set as one of the major indicators of the suitability of the culture system, with relevance to drug screening for loiasis. From values obtained after testing each system on three batches of parasites, T_90_ values were expressed as mean ± standard deviation.

The Kruskal-Wallis test was used to assess the global significant differences between the distribution of the median T_90_ across media and supplements, and the pairwise multiple comparisons of the ranked data was performed using the Pairwise Multiple Comparisons of Mean Rank (*PCMR*) package in R version 3.1.4. Mann-Whitney U-test was used to compare the the medians of the T_90_ between the cell free and cell containing culture. Statistical tests were interpreted using a 5% significance level. It was considered that a valid appreciation of the effect of any drug could be possible only in a system where at least 90% of parasites motility were sustained till the end of the experiment.

Factors that promoted parasite survival were identified using the multiple linear regression. The general linear model (GLM) was built using the hierarchical stepwise method. A total of 5 blocks were achieved with the 5 factors (incubation time, presence of feeder cells, basic medium, serum/protein, protein concentration) and those that contributed significantly to the improvement of the model were identified based on the *F*-statistics and the adjusted *R*-square (Additional file 1: Table S1). The incubation time was treated as a metric factor. Dichotomous variables such as the presence of monkey kidney cells were coded using binary figures. For each nominal or ordinal factor (Basic culture media, protein or protein concentration), sets of dummy variables were created and compared to one of the categories defined as reference. While RPMI-1640 was used as a reference against DMEM and IMDM, the four sera (Albumax II, BSA, FBS and NCS) were compared to the serum free culture (No serum). The three concentrations of each serum (0.5%, 1% and 1.5% for Albumax II and BSA; 5%, 10% and 15% for FBS and NCS) were labelled using the three ordinal levels: low, medium and high concentration; they were also compared to the serum-free culture. Interaction factors were created between explanatory variables and added to the models. The prediction of the motility by the protein concentration was poor, and there was a non-statistical difference between different concentrations of protein as will be discussed latter. Based on experimental observations, interactions were expected between the other three variables. Therefore, two ways and three ways interaction terms were created between those three experimental parameters: the presence of feeder cells, the culture medium used in reference to RPMI1640 and the type of protein supplement.

The passage of the *L. loa* larvae from the third (L3) to the fourth (L4) stages was further considered the second target product profile in assessing the suitability of the culture systems tested. For each of the 78 culture systems designed and evaluated, the moulting rate (percentage of moulted worms) as well as the timeframe was computed.

## Results

### Evaluation of the effect of each basic culture medium on the viability of *L. loa* L3 and mf

**Fig. 1:**
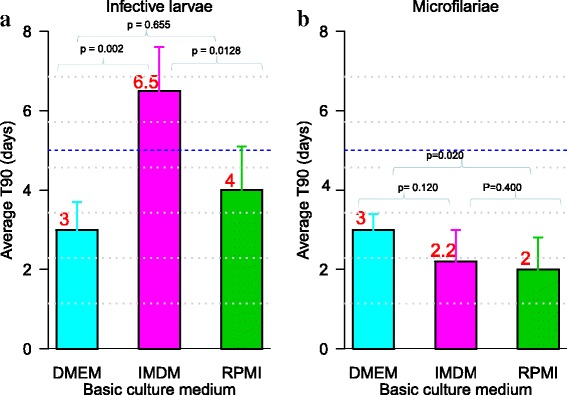
Effect of each basic medium on the mean values of T_90_ of *L. loa* L3 (**a**) and microfilarae (**b**). Microfilariae and infective larvae of *Loa loa* were cultured with three different media (RPMI, DMEM and IMDM) without neither serum supplement, nor feeder cells. Pairwise multiple comparisons: Dunn’s *post-hoc* test for multiple comparisons of independent samples. The *P-*values indicated here are those that were found significant when comparing two basic media. Number of observations: *n* = 12. *Abbreviations*: DMEM, Dulbecco’s modified Eagle’s medium; IMDM, Iscove’s modified Dulbecco’s medium; RPMI, Roosevelt Memorial Park Institute; L3, third-stage infective larva; T_90_, mean duration at which 90% of parasites were fully active

The T_90_ values of the parasite motility in different basic media were evaluated and results are presented in Fig. 1 and the statistical report in Table 1. The values of T_90_ ranged from 3.0 day to 6.5 days for L3. In the absence of protein supplement and feeder cells, there were significant difference between the T_90_ value of the 3 media vis a vis the L3 (*χ*^2^ = 38.793, *df* = 2, *P* < 0.0001) and mfs (*χ*^2^ = 38.793, *df* = 2, *P* < 0.0001). Pairwise comparison indicated that L3 survived longer particularly in IMDM compared to DMEM (*P* < 0.0001) and RPMI (*P* < 0.0001), whereas mf hardly exceeded 3 days survival, irrespective of the culture medium tested in absence of supplements. However, mf survival time was significantly higher in DMEM than in RPMI (*P* < 0.0001) or IMDM (*P* = 0.0010).

**Table 1:** Statistical report on the effect of serum/protein concentration in various culture systems with regards to the mean values of T_90_ of *L. loa* microfilariae and L3

Parasite	Feeder layer	Medium	Protein	Kruskal-Wallis *χ*^2^	*df*	*P*-value
*Loa loa* mf	No	DMEM	Albumax	4.2585	3	0.2349
			BSA	9.1681	3	0.0271
			FBS	9.6542	3	0.0218
			NCS	9.5639	3	0.0227
		IMDM	Albumax	16.1960	3	0.0010
			BSA	22.8860	3	< 0.0001
			FBS	19.1060	3	0.0003
			NCS	23.1030	3	< 0.0001
		RPMI	Albumax	20.5860	3	0.0001
			BSA	17.1480	3	0.0007
			FBS	7.5795	3	0.0556
			NCS	11.3590	3	0.0099
	LLC-MK2	DMEM	Albumax	6.9561	3	0.0733
			BSA	6.1987	3	0.1023
			FBS	4.9629	3	0.1745
			NCS	6.1198	3	0.1059
		IMDM	Albumax	0.7759	3	0.8552
			BSA	1.7466	3	0.6266
			FBS	3.0427	3	0.3851
			NCS	0.8119	3	0.8466
		RPMI	Albumax	16.8520	3	0.0008
			BSA	14.6670	3	0.0021
			FBS	0.3286	3	0.9546
			NCS	2.3611	3	0.5009
*Loa loa* L3	No	DMEM	Albumax	7.0250	3	0.0711
			BSA	8.3441	3	0.0394
			FBS	8.1322	3	0.0434
			NCS	6.0725	3	0.1081
		IMDM	Albumax	1.7999	3	0.6150
			BSA	1.8722	3	0.5993
			FBS	3.8436	3	0.2789
			NCS	9.6063	3	0.0222
		RPMI	Albumax	8.4292	3	0.0379
			BSA	4.2135	3	0.2393
			FBS	6.5292	3	0.0885
			NCS	1.4905	3	0.6845
	LLC-MK2	DMEM	Albumax	1.8177	3	0.6111
			BSA	1.3618	3	0.7145
			FBS	5.8987	3	0.1166
			NCS	1.6916	3	0.6388
		IMDM	Albumax	16.0890	3	0.0011
			BSA	5.2627	3	0.1535
			FBS	2.1496	3	0.5419
			NCS	4.9566	3	0.1750
		RPMI	Albumax	6.2386	3	0.1006
			BSA	5.5548	3	0.1354
			FBS	4.0227	3	0.2590
			NCS	5.6006	3	0.1327

### Evaluation of the effect of serum/protein supplementation on the viability of *L. loa* larvae and microfilariae in culture

**Fig. 2:**
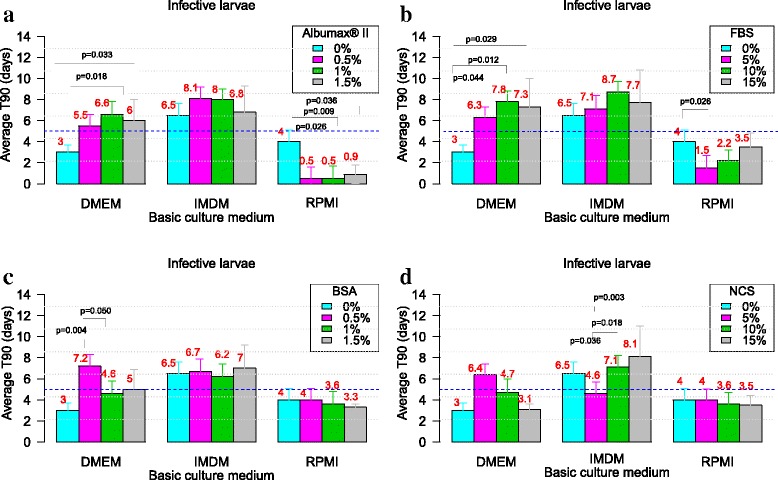
Effect of serum/protein supplements in various basic media with regards to the mean values of T_90_ of *L. loa* L3. Infective larvae of *Loa loa* were cultured with three different media (RPMI, DMEM and IMDM) with each of the four serum/protein supplements [Albumax (**a**), FBS (**b**), BSA (**c**) and NCS (**d**)] without feeder cells. Pairwise multiple comparisons: Dunn’s *post-hoc* test for multiple comparisons of independent samples. The *P-*values indicated here are those that were found significant when comparing two concentrations of the serum/protein. Number of observations: *n* = 12. *Abbreviations*: DMEM, Dulbecco’s modified Eagle’s medium; IMDM, Iscove’s modified Dulbecco’s medium; RPMI, Roosevelt Memorial Park Institute; BSA, bovine serum albumin; FBS, fetal bovine serum; NCS, newborn calve serum; L3, third-stage infective larva; T_90_, mean duration at which 90% of parasites were fully active

**Fig. 3:**
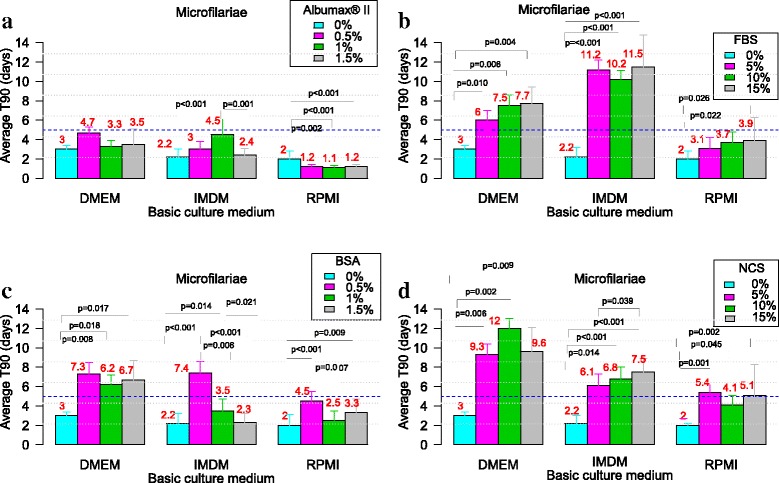
Effect of serum/protein supplements in various basic media with regards to the mean values of T_90_ of *L. loa* microfilariae. Microfilariae of *Loa loa* were cultured with three different media (RPMI, DMEM and IMDM) with each of the four serum/protein supplements [Albumax (**a**), FBS (**b**), BSA (**c**) and NCS (**d**)] without feeder cells. Pairwise multiple comparisons: Dunn’s *post-hoc* test for multiple comparisons of independent samples. The *P-*values indicated here are those that were found significant when comparing two concentrations of the serum/protein. Number of observations: *n* = 12. *Abbreviations*: DMEM, Dulbecco’s modified Eagle’s medium; IMDM, Iscove’s modified Dulbecco’s medium; RPMI, Roosevelt Memorial Park Institute; BSA, bovine serum albumin; FBS, fetal bovine serum; NCS, newborn calve serum; T_90_: mean duration at which 90% of parasites were fully active

Figure 2 and Fig. 3 show the effect of serum/protein supplements in various basic media with regards to the mean values of T_90_ of *L. loa* L3 and mf, respectively.

### Effect of serum/protein on the viability of *L. loa* larvae

#### AlbuMax® II

Generally, the supplementation of DMEM and IMDM with AlbuMax® II promoted the viability of larvae, increasing T_90_ by 1.5–2.5-fold. RPMI supplemented with AlbuMax® II inhibited the larvae viability (T_90_ < 1 day, Fig. 2a).

#### Fetal bovine serum (FBS)

This serum could support the viability of larvae for up to 8.7 days when IMDM was supplemented with 10% FBS. T_90_ > 5 days was observed in all concentrations of FBS except in the RPMI supplement medium (Fig. 2b).

#### Bovine serum albumin (BSA)

All media supplemented with BSA also improved larvae viability with T_90_ ≥ 5 days, except for DMEM supplemented with 1% BSA and all concentrations of BSA with RPMI (Fig. 2c).

#### Newborn calf serum (NCS)

Considering media supplemented with NCS, only DMEM with 5% NCS, IMDM with 10/15% NCS had T_90_ > 5 days (Fig. 2d). Considering the 5-day cut-off point for drug screening, up to twenty culture media formulated based on the two basic culture media (DMEM and IMDM) and the four serum/protein supplements can be exploited for *Loa* L3 in priority: DMEM with 0.5% AlbuMax® II (T_90_ = 5.5 ± 1.9), 1% AlbuMax® II (T_90_ = 6.6 ± 2.4), 1.5% AlbuMax® II (T_90_ = 6 ± 1.8), 0.5% BSA (T_90_ = 7.2 ± 0.3), 1.5% BSA (T_90_ = 5 ± 1.9), 5% FBS (T_90_ = 6.3 ± 2), 10% FBS (T_90_ = 7.8 ± 2.2), 5% FBS (T_90_ = 7.3 ± 2.7), 5% NCS (T_90_ = 6.4 ± 2.5), and IMDM with 0.5% AlbuMax® II (T_90_ = 8.5 ± 3.8), 1% AlbuMax® II (T_90_ = 8 ± 2.5), 1.5% AlbuMax® II (T_90_ = 6.8 ± 2.5), 0.5% BSA (T_90_ = 6.7 ± 1.2), 1% BSA (T_90_ = 6.2 ± 1.5), 1.5% BSA (T_90_ = 7 ± 2.2), 5% FBS (T_90_ = 7.1 ± 3), 10% FBS (T_90_ = 8.7 ± 2.6), 15% FBS (T_90_ = 7.7 ± 3.1), 10% NCS (T_90_ = 7.1 ± 2.4), 15% NCS (T_90_ = 8.1 ± 2.9).

### Effect of serum/protein on the viability of *L. loa* microfilariae

#### AlbuMax® II

All concentrations of AlbuMax® II supplement improve the viability of mf regardless the basic medium except for RPMI. Although the improvement was noticeable, all T_90_ were less than 5 days as shown in Fig. 3a.

#### Fetal bovine serum (FBS)

All concentrations of fetal bovine serum improved parasite viability but T_90_ > 5 days was reported only with FBS supplemented DMEM and IMDM (Fig. 3b).

#### Bovine serum albumin (BSA)

Generally, BSA boosted the microfilariae viability. The T_90_ values above 5 days were reported with all concentrations of BSA supplemented DMEM and IMDM + 0.5% BSA (Fig. 3c).

#### Newborn calf serum (NCS)

With respect to media supplemented with NCS, only RPMI supplemented with 10% NCS could not sustain the *L*. *loa* mf for up to T_90_ = 5 days. Generally, NCS supplementation improved the *L*. *loa* mf viability by up to 4-fold as compared to basic medium without protein supplement (DMEM with 10%NCS, T_90_ = 12 ± 1 days and DMEM only, T_90_ = 3 ± 0.4 days), respectively (Fig. 3d).

With respect to *Loa* mf, none of the media supplemented with AlbuMax® II irrespective of the protein concentration could improve microfilaria viability for up to T90 ≥ 5. Nevertheless, up to eighteen culture media formulations based on all three basic culture media and the four serum/protein supplements had interesting T_90_ values. These are DMEM with 0.5% BSA (T_90_ = 7. 3 ± 1.8), 1% BSA (T_90_ = 6.2 ± 1.4), 1.5% BSA (T_90_ = 6.7 ± 1.7), 5% FBS (T_90_ = 6 ± 3.1), 10% FBS (T_90_ = 7.5 ± 2.9), 15% FBS (T_90_ = 7.7 ± 1.7), 5% NCS (T_90_ = 9.3 ± 4.4), 10% NCS (T_90_ = 12 ± 1), 15% NCS (T_90_ = 9.6 ± 2.5**)**; IMDM with 0.5% BSA (T_90_ = 7.4 ± 3.1), 5% FBS (T_90_ = 11.2 ± 2.6), 10% FBS (T_90_ = 10.2 ± 1.9), **1**5% FBS (T_90_ = 11.5 ± 3.3), 5% NCS (T_90_ = 6.1 ± 1.2), 10% NCS (T_90_ = 6.8 ± 1.2), 15% NCS (T_90_ = 7.5 ± 1.4) and RPMI with 5% NCS (T_90_ = 5.4 ± 4.9), 15% NCS (T_90_ = 5.1 ± 3.2).

### Assessment of the importance of monkey kidney cells as feeder layer

**Fig. 4:**
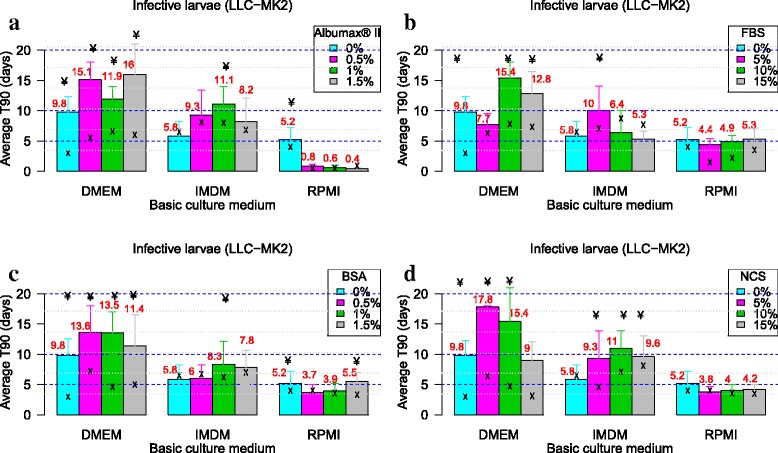
Effect of monkey kidney cells in various serum/protein supplemented basic media with regards to the mean values of T_90_ (days) of *L. loa* L3. The black arrows indicate the T_90_ values in absence of the feeder cells. Infective larvae of *Loa loa* were cultured with three different media (RPMI, DMEM and IMDM) with each of the four serum/protein supplements [Albumax (**a**), FBS (**b**), BSA (**c**) and NCS (**d**)] and (LLC-MK2) feeder cells. For each medium and each concentration of the serum/protein, culture with monkey kidney cells was compared to serum free culture (Mann-Whitney U-test). Number of observations: *n* = 12. *Abbreviations*: DMEM, Dulbecco’s modified Eagle’s medium; IMDM, Iscove’s modified Dulbecco’s medium; RPMI, Roosevelt Memorial Park Institute; BSA, bovine serum albumin; FBS, fetal bovine serum; NCS, newborn calve serum; L3, third-stage infective larva; T_90_, mean duration at which 90% of parasites were fully active; ¥: significantly different from the equivalent cell free culture

**Fig. 5:**
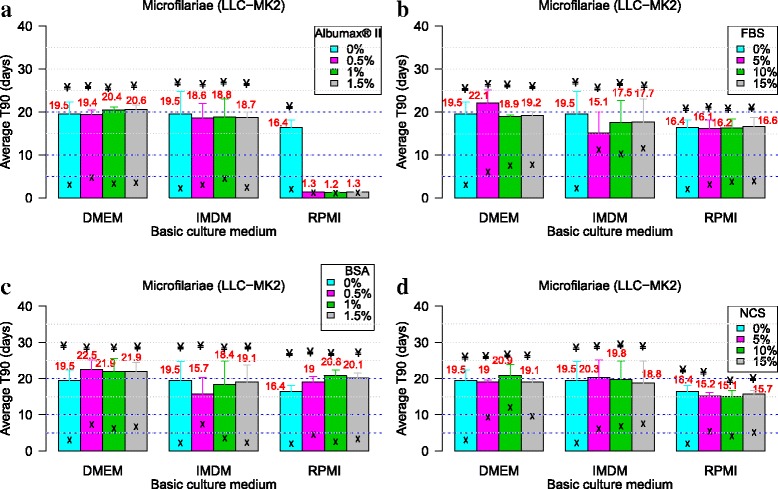
Effect of monkey kidney cells in various serum/protein supplemented basic media with regards to the mean values of T_90_ of *L. loa* microfilariae. Microfilariae of *Loa loa* were cultured with three different media (RPMI, DMEM and IMDM) with each of the four serum/protein supplements [Albumax (**a**), FBS (**b**), BSA (**c**) and NCS (**d**)] and (LLC-MK2) feeder cells. For each medium and each concentration of the serum/protein, culture with monkey kidney cells was compared to serum free culture (Mann-Whitney U-test). Number of observations: *n* = 12. *Abbreviations*: DMEM, Dulbecco’s modified Eagle’s medium; IMDM, Iscove’s modified Dulbecco’s medium; RPMI, Roosevelt Memorial Park Institute; BSA, bovine serum albumin; FBS, fetal bovine serum; NCS, newborn calve serum; T_90_, mean duration at which 90% of parasites were fully active; ¥, significantly different from the equivalent cell free culture

The findings on the effect of the monkey kidney epithelial cells as feeder on the survival of the *L. loa* L3 and mf are summarized in Fig. 4 and Fig. 5, respectively and the summary of the statistical report is presented in Table 2. Co-culture of L3 or mf with LLC-MK2 cells significantly improved the longevity of parasites culture with each basic media in the absence of serum/protein supplements. With respect to *Loa* L3, only DMEM with LLC-MK2 (No serum/protein supplement) enhanced viability by 3-fold (9.8 ± 2.7), as compared to DMEM without serum (T_90_ of 3 ± 1). It was not the case regarding IMDM with LLC-MK2 (T_90_ = 5.8 ± 2.4) and RPMI with LLC-MK2 (T_90_ = 5.2 ± 2). When combining feeder cells with serum/protein supplements, the parasite survival and motility were even more increased, with highest T_90_ per basic culture medium varying from 10.0 (5% FBS in IMDM) to 17.8 days (5% NCS in DMEM).

**Table 2:** Summary statistics on the effect of the addition of monkey kidney cells in various serum/protein supplemented basic media with regards to the mean values of T_90_ of *L. loa* L3 and microfilariae

Medium	Serum/ Protein	Concentration (%)	*Loa loa* L3		*Loa loa* mf	
			Mann-Whitney *U*	*P-*value	Mann-Whitney *U*	*P-*value
DMEM	No serum		32	0.0084	24	0.0139
	Albumax	0.5	16	0.0452	12	0.0471
		1	16	0.0476	35	0.0025
		1.5	21	0.0467	16	0.0286
	BSA	0.5	15	0.0167	12	0.0471
		1	19	0.0171	48	0.0006
		1.5	14	0.0462	32	0.0040
	FBS	5	6	0.5462	15	0.0357
		10	15	0.0167	24	0.0095
		15	12	0.0448	35	0.0025
	NCS	5	15	0.0167	6	0.0200
		10	22	0.0381	20	0.0159
		15	24	0.0095	20	0.0158
IMDM	No serum		25	0.4945	56	0.0003
	Albumax	0.5	20.5	0.7479	54	0.0004
		1	36	0.0137	80	0.0004
		1.5	21.5	0.9999	70	0.0001
	BSA	0.5	14.5	0.6303	36	0.0069
		1	32	0.0345	96	0.0002
		1.5	25	0.9497	96	0.0002
	FBS	5	26	0.024	18	0.0257
		10	12	0.1419	91	0.0003
		15	11	0.1079	72	0.0490
	NCS	5	31	0.0411	54	0.0004
		10	38	0.0813	96	< 0.0001
		15	31	0.0414	96	< 0.0001
RPMI	No serum		26	0.0109	98	0.0003
	Albumax	0.5	14	0.2413	32.5	0.3170
		1	20	0.1087	83	0.0685
		1.5	10	0.7483	86	0.1764
	BSA	0.5	6	0.9999	40	0.002
		1	9	0.4000	77	< 0.0001
		1.5	17	0.0111	77	0.0006
	FBS	5	15	0.3571	35	0.0057
		10	16	0.2857	56	< 0.0001
		15	16	0.1905	91	0.0004
	NCS	5	6.5	0.9999	29	0.0282
		10	9	0.4000	88	0.0003
		15	13	0.2000	88	0.0003

Co-culture of microfilariae with feeder cells increased T_90_ values up to 7-fold (22.5 ± 2.7 days). All serum/protein supplemented systems in co-culture were suitable (T_90_ significantly greater than 5 days) with DMEM and IMDM; but RPMI supplemented with AlbuMax® II remained sub-standard despite the presence of LLC-MK2 feeder layer. Microfilariae co-cultured on LLC-MK2 in basic culture media alone T_90_ values (DMEM T_90_ = 19.5 ± 2.8; IMDM T_90_ = 19.5 ± 5.2; and RPMI T_90_ = 16.4 ± 1.7) were not statistically different from their best protein/serum supplemented counterpart (0.5% BSA supplemented DMEM on LLC-MK2 T_90_ = 22.5 ± 2.7; 5% NCS supplemented IMDM on LLC-MK2 T_90_ = 20.3 ± 4.9; and 1% BSA supplemented RPMI on LLC-MK2 T_90_ = 20.8 ± 1.6). The ranking of the observed T_90_ values of the various tested systems are summarised in Additional file 2: Table S2.

### Effect of the different culture systems on the moulting from L3 to the fourth-stage larvae of *L. loa*

**Fig. 6:**
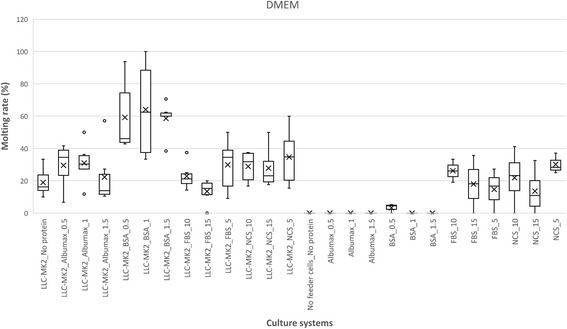
Effects of serum/protein supplements at different concentrations added to DMEM basic medium with or without feeder layer on the moulting *L. loa* infective larvae in culture. Number of observations: *n* = 12. *Abbreviation*: DMEM: Dulbecco’s modified Eagle’s medium

**Fig. 7:**
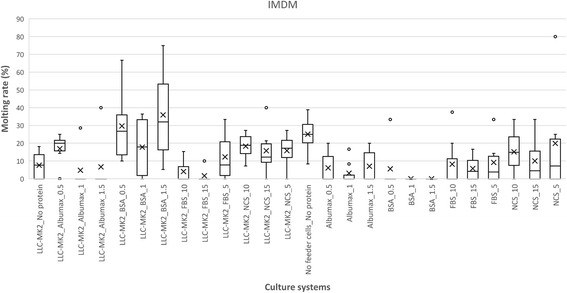
Effects of serum/protein supplements at different concentrations added to IMDM basic medium with or without feeder layer on the moulting *L. loa* infective larvae in culture. Number of observations: *n* = 12. *Abbreviation*: IMDM: Iscove’s modified Dulbecco’s medium

**Fig. 8:**
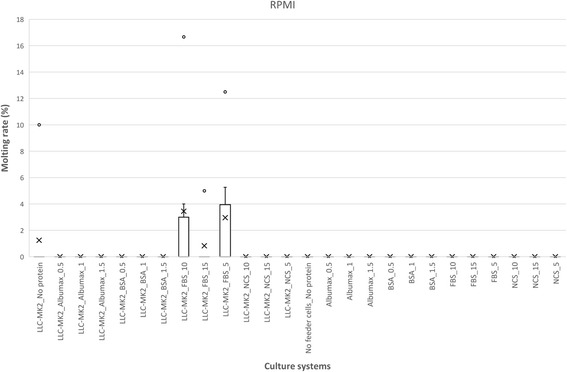
Effects of serum/protein supplements at different concentrations added to RPMI basic medium with or without feeder layer on the moulting *L. loa* infective larvae in culture. Number of observations: *n* = 12. *Abbreviation*: RPMI: Roosevelt Memorial Park Institute

**Fig. 9:**
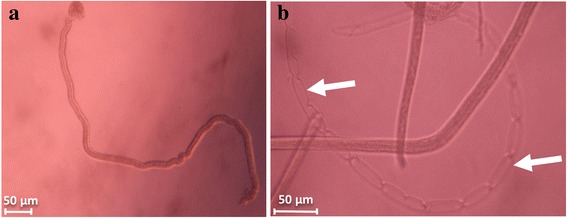
Image of moulted worms observed under inverted microscope. **a** Fully moulted *Loa* L4 larva. **b** Cast cuticule from moulted *Loa* L3 (arrows)

Moulting was observed following *L. loa* L3 culture, but its occurrence varied widely with culture conditions with values up to 70.37% (in 1% BSA supplemented DMEM in co-culture with LLC-MK2). Variation in moulting rate are presented in Figs. 6, 7 and 8, Additional file 3: Table S3, with an illustration in Fig. 9. The proportion of moulting recorded with RPMI was below 25%. This contrasted with DMEM and IMDM where significant moulting rates were noted, both in cell-free and co-culture of L3 with LLC-MK2. Apart from the isolated case of IMDM with up to 27.27% (24 moulted worms out of a total of 88), serum/protein supplementation was found indispensable for the transition from L3 to L4 *in vitro*. The higher moulting rates were generally observed with DMEM, compared to IMDM. The highest moulting rate was observed in protein and feeder layer supplemented DMEM as illustrated in Fig. 6. *Loa loa* L3 moulting began on day 9 and observation was continued until day 29, supplementation with BSA scored highest moulting rates, with 57.32, 70.37 and 58.62% larvae moulted in 0.5, 1 and 1.5% BSA supplement, respectively.

### Linear regression analysis of different factors that influence the *in vitro* maintenance of *L. loa* microfilariae and L3

**Fig. 10:**
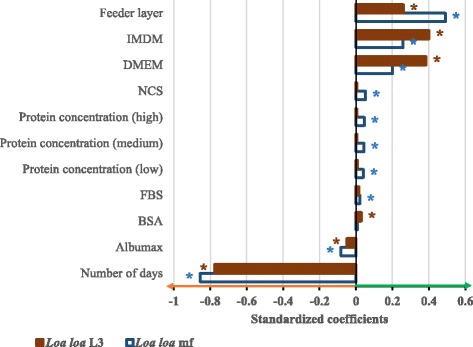
Graphical representation of the standardized coefficients of the main effects of different factors on the predicted *Loa loa* microfilariae and L3 motility. Adjusted *R*^2^: *L. loa* mf = 0.709; *L. loa* L3 = 0.716

Bivariate analysis indicated strong association between motility and incubation time (Spearman’s rho = -0.674, *P* < 0.001). This was the first to be introduced in the single linear regression analysis (*R*^2^ = 0.452), and the remaining variables were successively added to construct the final GLM. Before interaction terms were introduced in the models, the important factors that contributed to the improvement of worm motility were identified separately based on their standardized coefficient (Fig. 10). For *Loa loa* mf, these factors included feeder cells (*β* = 0.490), both IMDM (*β* = 0.256) and DMEM (*β* = 0.198) media and the protein supplements NCS (*β* = 0.052) and FBS (*β* = 0.022); for *Loa loa* L3, in addition to feeder cells (*β* = 0.259) and both IMDM (*β* = 0.401) and DMEM (*β* = 0.385) media, the proteins supplement BSA (*β* = 0.029) were found important for the maintenance of the worm motility.

From the built models, the important variables required to meet the threshold of T_90_ are as followed and classified with respect to their importance. Feeder cells were found as the most important. In combination to DMEM (DMEM-LLCMK2), the unstandardized coefficient decreased although the interaction was the best among others. In addition, the combination of three variables (feeder layer-basic culture medium-protein/serum supplement) was not mandatory as the interaction LLCMK2-Albumax instead weakened the model with a negative coefficient.

The model was diagnosed by assessing the assumptions of normal distribution and homoscedasticity. The histogram of the residuals (errors) in the model was used to check if they are normally distributed (Additional file 4: Figure S1). Although not perfect, the frequency distribution of the residuals has a shape close to that of the normal Gauss curve, indicating evidence of normal distribution. Additionally, P-P plot was used for further check (Additional file 5: Figure S2). Here, the expected and observed cumulative probabilities were closed suggesting that the assumption of normal distribution of the residual was far to be not violated. The scatterplot of standardized residuals against standardized predicted values were used to assess the assumption of homoscedasticity (Additional file 6: Figure S3). The variance of residuals were random distributed indicating that the assumption of homoscedasticity was likely to be safe.

## Discussion

Drug discovery research for *L*. *loa* has so far attracted only very limited attention compared to other filarial diseases. Repurposing attempts have been conducted with existing drugs with only limited success. The effect of several antimalarial drugs (quinine, chloroquine, amodiaquine and artesunate) was investigated on loiasis in a randomized, placebo-controlled approach in central Cameroon [20]. This study recorded no significant change in parasite loads in any of the treatment groups. Another study tested different intermittent doses of albendazole on Loa loa microfilaraemia, the reduction in mf load obtained was insufficient to prevent the risk of severe adverse reactions during ivermectin mass drug administration in loiasis co-endemic areas [21]. These observations demonstrated that repurposing of existing antiparasitic therapies may not be a suitable approach to develop drugs with satisfactory therapeutic window. The traditional approach starting from standard *in vitro* discovery through preclinical and clinical testing necessitates *in vitro* maintenance of *L*. *loa* stages for a minimum duration required for drug screening. Herein we designed and tested the effect of varying 78 culture conditions on both L3 and mf viability starting with three basic culture media, four serum/protein supplements and one feeder cell. In general, *L*. *loa* L3 survived for longer periods than mf in the different basic culture media with neither protein nor feeder cell supplementation. For the maintenance of L3, IMDM exhibited the best performance, whereas on microfilariae, DMEM had the highest T_90_ though only three-day survival. Considering a minimum cut-off point of five-day maintenance of 90% highly active larvae or microfilariae for *in vitro* drug screening on *L*.* loa* microfilariae and infective larvae, IMDM was the only basic medium that could be employed as such without any need of protein supplement/feeder layer. However, none of the media would be suitable for *in vitro* investigations requiring longer periods of incubation.

Four different serum/protein supplements were therefore applied at increasing concentrations to the three basic culture media in order to improve their nutritional potencies for both L3 and mf. The results obtained were highly diverse. In RPMI, serum/protein supplementation rather caused drop in T_90_ which was more pronounced for AlbuMax® II followed by FBS, NCS and BSA, in both L3 and mf. Consequently, none of the formulations based on RPMI supplemented with serum/protein (without feeder layer) was successful in keeping mf alive and active for more than five days. Mengome et al. [22] recently reported on screening of 12 methanolic extracts of nine traditional plant remedies employed in Gabon, on *L*. *loa* mf maintained *in vitro* using modified Eagle’s medium supplemented with 10% foetal calf serum with five-day incubation time. Our findings showing an average T_90_ value of 7.5 ± 2.9 days and thus corroborate the data reported by these authors, confirming the suitability of the culture system employed.

A drastic increase in the survival time and viability of the *L. loa* mf was obtained with addition of LLC-MK2 cells as feeder layer, except for AlbuMax® II in RPMI. The T_90_ values were extended for all culture formulations (except AlbuMax® II in RPMI), even exceeding 20 days.

The hypothesis that LLC-MK2 cells alone can be enough to sustain the viability of the *L. loa* parasites *in vitro* was tested. In all the three basic culture media, RPMI, DMEM and IMDM, without serum/protein supplement, parasites could still survive 15 days and beyond. Toback et al. [23] reported that monkey kidney cells during their growth *in vitro* produce growth factors such as the epidermal growth factor (EGF), interleukin growth factor (IGF) and transforming growth factor (TGF-β). Thus, these factors are possibly supportive of *L. loa* larval growth and/or survival, which facilitate their *in vitro* maintenance.

Culture systems capable of sustaining moulting of infective larvae present an additional advantage for *in vitro* investigations on the parasite, including both physiological studies and the exploration of drug targets. In addition to the survival of parasites, the effect of the different culture systems on the moulting of L3 was further examined. Maintenance and moulting of filariae larvae using different culture systems have been reported for *Onchocerca* spp. [24, 25], *Wuchereria bancrofti* [26–29] and *Brugia malayi* [30, 31]. The present study is the first attempt to optimise such systems for *L. loa* larvae.

Of the total of 659 moulted worms observed, the distribution with respect to each medium was as followed: DMEM (70%) followed by IMDM (29%) and finally RPMI (1%). The proportion of moulting observed in DMEM + LLC-MK2 varied with the nature of the serum/protein supplement. 62.44% (138/221) in BSA, 30.15% (79/262) in NCS, 24.52% (64/261) in AlbuMax® II and 22.38% (45/201) in FBS. The moulting rate in BSA was statistically different (*P* < 0.001) from the three other sera. With BSA supplementation, 1% proved to be optimum with a 70.37% moulting rate, compared with 1.5% and 0.5% with rates of 58.62% and 57.32%, respectively. Although the role of albumin in the promotion of *L. loa* L3 moulting is still to be elucidated, Ishima et al. [32] reported on some beneficial effects from the interaction of albumin with other biological factors such as insulin, epidermal growth factor in the *in vitro* culture of mammalian cells, suggesting that this combination of BSA and LLC-MK2 as supplements provided optimal *in vitro* conditions for *L. loa* L3 moulting. Smith et al. [33] have developed a serum-free *in vitro* system for *Brugia malayi* third infective larvae, by supplementing RPMI 1640 with either arachidonic, linoleic or linolenic acids and this supported consistent and reproducible moulting to the fourth larval stage in the presence of a basidiomycetous yeast, *Rhodotorula minuta*. In serum-free cultures lacking *R. minuta*, L3 larvae survive for upward of two weeks, but did not moult. Smith & Rajan [34] subsequently used this system to study the effect of tetracycline on three different species of filarial nematodes, *Brugia malayi*, *Brugia pahangi* and *Dirofilaria immitis*.

In summary, IMDM and DMEM were the two basic media found to be more suitable to culture *L. loa* L3 and mf, respectively, for short incubation time (for up to 7 days); DMEM + 5% NCS and IMDM + 10% FBS are suitable to culture both *L. loa* mf and L3 for relatively long incubation time (for up to two weeks); DMEM + LLC-MK2 was suitable to culture *L. loa* mf for long incubation time while DMEM + 1% BSA + LLC-MK2 provided optimal moulting conditions for *L. loa* L3.

## Conclusions

This study has demonstrated the effects of protein supplemented basic media in association with or without monkey kidney cells on the survivorship of *L. loa* microfilariae and on the survival and moulting of *L. loa* infective larvae. The findings from this work provide a range of culture requirements for the maintenance of *L. loa*, which are suitable for developing an effective platform for drug screening.

## Additional files

Additional file 1: **Table S1.** Summary of the contribution of the main effects of various variables in the model. (DOCX 26 kb)

Additional file 2: **Table S2.** Ranking of the various experimental systems. (DOCX 26 kb)

Additional file 3: **Table S3.** Moulting rate (%) of *L. loa* L3 in different *in vitro* culture systems. (DOCX 17 kb)

Additional file 4: **Figure S1.** Motility standardized residual histogram of the motility. (DOCX 41 kb)

Additional file 5: **Figure S2.** Gaussian regression P-P plot of predicted motility. (DOCX 27 kb)

Additional file 6: **Figure S3.** Scatterplot of standardized residuals against standardized predicted values. (DOCX 29 kb)

## Abbreviations

APOC: African Programme for Onchocerciasis Control
BSA: bovine serum albumin
DMEM: Dulbecco’s modified Eagle’s medium
FBS: foetal bovine serum
IMDM: Iscove’s modified Dulbecco’s medium
LLC-MK2: Lewis lung carcinoma Monkey Kidney cell line 2
NCS: newborn calf serum
NTDs: neglected tropical diseases
RPMI: Roswell Park Memorial Institute
T_90_: mean duration (days) at which at least 90% of worms were fully active
