# Supplementary material for: Evaluation of in vitro culture systems for the maintenance of microfilariae and infective larvae of Loa loa
Source: Parasit Vectors. 2018 May 2;11:275. doi: 10.1186/s13071-018-2852-2 (PMC5930665; doi:10.1186/s13071-018-2852-2)
Supplement: Supplementary file 1 — Table S1. Summary of the contribution of the main effects of various variables in the model. (DOCX 26 kb) [file 13071_2018_2852_MOESM1_ESM.docx]

| **Additional file 1: Table S1.** Summary of the contribution of the main effects of various variables in the model | | | | | | | | | | |
| --- | --- | --- | --- | --- | --- | --- | --- | --- | --- | --- |
| **Stages** | **Model** | **R** | **R^2^** | **adjusted R^2^** | **Standard error of the estimate** | **Change in statistics** | | | | |
|  |  |  |  |  |  | **Variation of R^2^** | **Variation of F** |  |  | **Sig. F variation** |
| ***Loa* mf** | **a** | 0.643a | 0.414 | 0.414 | 33.25879 | 0.414 | 9661.411 |  |  | <0.001 |
|  | **b** | 0.799b | 0.639 | 0.639 | 26.10140 | 0.225 | 8545.266 |  |  | <0.001 |
|  | **c** | 0.833c | 0.694 | 0.694 | 24.03480 | 0.055 | 1229.615 |  |  | <0.001 |
|  | **d** | 0.841d | 0.708 | 0.707 | 23.49196 | 0.014 | 161.092 |  |  | <0.001 |
|  | **d** | 0.842^e^ | 0.709 | 0.709 | 23.41989 | 0.002 | 29.137 |  |  | <0.001 |
| ***Loa* L3** | **a** | 0.734a | 0.539 | 0.539 | 26.04119 | 0.539 | 11012.353 |  |  | <0.001 |
|  | **b** | 0.766b | 0.587 | 0.587 | 24.63945 | 0.048 | 1103.410 |  |  | <0.001 |
|  | **c** | 0.844c | 0.712 | 0.711 | 20.60096 | 0.124 | 2028.642 |  |  | <0.001 |
|  | **d** | 0.846d | 0.716 | 0.716 | 20.43609 | 0.005 | 39.143 |  |  | <0.001 |
|  | **e** | **0.846e** | **0.716** | **0.716** | **20.43751** | **0.000** | **0.564** |  |  | 0.639 |
|  |  |  |  |  |  |  |  |  |  |  |

a. Predicted values : (constants), Days

b. Predicted values : (constants), Days, Feeder layer

c. Predicted values : (constants), Days, Feeder layer, Medium concentration, DMEM, IMDM

d. Predicted values : (constants), Days, Feeder layer, DMEM, IMDM, FBS, NCS, BSA, Albumax

e. Predicted values : (constants), Number of days, Feeder layer, DMEM, IMDM, FBS, NCS, BSA, Albumax, Low concentration, Medium concentration, High concentration,

Dependent variable : Motility
