# Supplementary material for: Evaluation of in vitro culture systems for the maintenance of microfilariae and infective larvae of Loa loa
Source: Parasit Vectors. 2018 May 2;11:275. doi: 10.1186/s13071-018-2852-2 (PMC5930665; doi:10.1186/s13071-018-2852-2)
Supplement: Supplementary file 2 — Table S2. Ranking of the various experimental systems. (DOCX 26 kb) [file 13071_2018_2852_MOESM2_ESM.docx]

# Additional file 2: Table S2. Ranking of the various experimental systems

| *Ranking | *Loa loa* L3 | | *Loa loa* mf | |
| --- | --- | --- | --- | --- |
|  | System | Average of T90 (days) | System | Average of T90 (days) |
| 1 | DMEM+LLC-MK2+5%NCS | 17.8 | DMEM+LLC-MK2+0.5%BSA | 22.5 |
| 2 | DMEM+LLC-MK2+1.5%Albumax | 16.0 | DMEM+LLC-MK2+5%FBS | 22.1 |
| 3 | DMEM+LLC-MK2+10%FBS | 15.4 | DMEM+LLC-MK2+1%BSA | 21.9 |
| 4 | DMEM+LLC-MK2+10%NCS | 15.4 | DMEM+LLC-MK2+1.5%BSA | 21.9 |
| 5 | DMEM+LLC-MK2+0.5%Albumax | 15.1 | DMEM+LLC-MK2+10%NCS | 20.9 |
| 6 | DMEM+LLC-MK2+0.5%BSA | 13.6 | RPMI+LLC-MK2+1%BSA | 20.8 |
| 7 | DMEM+LLC-MK2+1%BSA | 13.5 | DMEM+LLC-MK2+1.5%Albumax | 20.6 |
| 8 | DMEM+LLC-MK2+15%FBS | 12.8 | DMEM+LLC-MK2+1%Albumax | 20.4 |
| 9 | DMEM+LLC-MK2+1%Albumax | 11.9 | IMDM+LLC-MK2+5%NCS | 20.3 |
| 10 | DMEM+LLC-MK2+1.5%BSA | 11.4 | RPMI+LLC-MK2+1.5%BSA | 20.1 |
| 11 | IMDM+LLC-MK2+1%Albumax | 11.1 | IMDM+LLC-MK2+10%NCS | 19.8 |
| 12 | IMDM+LLC-MK2+10%NCS | 11.0 | DMEM+LLC-MK2+No protein | 19.5 |
| 13 | IMDM+LLC-MK2+5%FBS | 10.0 | IMDM+LLC-MK2+No protein | 19.5 |
| 14 | DMEM+LLC-MK2+No protein | 9.8 | DMEM+LLC-MK2+0.5%Albumax | 19.4 |
| 15 | IMDM+LLC-MK2+15%NCS | 9.6 | DMEM+LLC-MK2+15%FBS | 19.2 |
| 16 | IMDM+LLC-MK2+5%NCS | 9.3 | DMEM+LLC-MK2+15%NCS | 19.1 |
| 17 | IMDM+LLC-MK2+0.5%Albumax | 9.3 | IMDM+LLC-MK2+1.5%BSA | 19.1 |
| 18 | DMEM+LLC-MK2+15%NCS | 9.0 | DMEM+LLC-MK2+5%NCS | 19.0 |
| 19 | IMDM+10%FBS | 8.7 | RPMI+LLC-MK2+0.5%BSA | 19.0 |
| 20 | IMDM+LLC-MK2+1%BSA | 8.3 | DMEM+LLC-MK2+10%FBS | 18.9 |
| 21 | IMDM+LLC-MK2+1.5%Albumax | 8.2 | IMDM+LLC-MK2+1%Albumax | 18.8 |
| 22 | IMDM+15%NCS | 8.1 | IMDM+LLC-MK2+15%NCS | 18.8 |
| 23 | IMDM+0.5%Albumax | 8.1 | IMDM+LLC-MK2+1.5%Albumax | 18.7 |
| 24 | IMDM+1%Albumax | 8.0 | IMDM+LLC-MK2+0.5%Albumax | 18.6 |
| 25 | IMDM+LLC-MK2+1.5%BSA | 7.8 | IMDM+LLC-MK2+1%BSA | 18.4 |
| 26 | DMEM+10%FBS | 7.8 | IMDM+LLC-MK2+15%FBS | 17.7 |
| 27 | DMEM+LLC-MK2+5%FBS | 7.7 | IMDM+LLC-MK2+10%FBS | 17.5 |
| 28 | IMDM+15%FBS | 7.7 | RPMI+LLC-MK2+15%FBS | 16.6 |
| 29 | DMEM+15%FBS | 7.3 | RPMI+LLC-MK2+No protein | 16.4 |
| 30 | IMDM+1.5%Albumax | 7.3 | RPMI+LLC-MK2+10%FBS | 16.2 |
| 31 | DMEM+0.5%BSA | 7.2 | RPMI+LLC-MK2+5%FBS | 16.1 |
| 32 | IMDM+10%NCS | 7.1 | IMDM+LLC-MK2+0.5%BSA | 15.8 |
| 33 | IMDM+5%FBS | 7.1 | RPMI+LLC-MK2+15%NCS | 15.7 |
| 34 | IMDM+1.5%BSA | 7.1 | RPMI+LLC-MK2+5%NCS | 15.2 |
| 35 | IMDM+0.5%BSA | 6.7 | IMDM+LLC-MK2+5%FBS | 15.1 |
| 36 | DMEM+1%Albumax | 6.6 | RPMI+LLC-MK2+10%NCS | 15.1 |
| 37 | IMDM+No protein | 6.5 | DMEM+10%NCS | 12.0 |
| 38 | IMDM+LLC-MK2+10%FBS | 6.4 | IMDM+15%FBS | 11.5 |
| 39 | DMEM+5%NCS | 6.4 | IMDM+5%FBS | 11.2 |
| 40 | DMEM+5%FBS | 6.4 | IMDM+10%FBS | 10.2 |
| 41 | IMDM+1%BSA | 6.2 | DMEM+15%NCS | 9.6 |
| 42 | DMEM+1.5%Albumax | 6.1 | DMEM+5%NCS | 9.3 |
| 43 | IMDM+LLC-MK2+0.5%BSA | 6.0 | DMEM+15%FBS | 7.7 |
| 44 | IMDM+LLC-MK2+No protein | 5.8 | DMEM+10%FBS | 7.5 |
| 45 | RPMI+LLC-MK2+1.5%BSA | 5.7 | IMDM+15%NCS | 7.5 |
| 46 | DMEM+0.5%Albumax | 5.5 | IMDM+0.5%BSA | 7.4 |
| 47 | RPMI+LLC-MK2+15%FBS | 5.3 | DMEM+0.5%BSA | 7.3 |
| 48 | IMDM+LLC-MK2+15%FBS | 5.3 | IMDM+10%NCS | 6.8 |
| 49 | RPMI+LLC-MK2+No protein | 5.2 | DMEM+1.5%BSA | 6.7 |
| 50 | RPMI+LLC-MK2+10%FBS | 5.0 | DMEM+1%BSA | 6.2 |
| 51 | DMEM+1.5%BSA | 5.0 | IMDM+5%NCS | 6.1 |
| 52 | DMEM+10%NCS | 4.7 | DMEM+5%FBS | 6.0 |
| 53 | DMEM+1%BSA | 4.6 | RPMI+5%NCS | 5.4 |
| 54 | IMDM+5%NCS | 4.6 | RPMI+15%NCS | 5.1 |
| 55 | RPMI+LLC-MK2+1%BSA | 4.5 | DMEM+0.5%Albumax | 4.8 |
| 56 | RPMI+LLC-MK2+15%NCS | 4.4 | IMDM+1%Albumax | 4.5 |
| 57 | RPMI+LLC-MK2+10%NCS | 4.3 | RPMI+0.5%BSA | 4.5 |
| 58 | RPMI+LLC-MK2+5%FBS | 4.2 | RPMI+10%NCS | 4.1 |
| 59 | RPMI+LLC-MK2+0.5%BSA | 4.1 | RPMI+15%FBS | 3.9 |
| 60 | RPMI+LLC-MK2+5%NCS | 4.1 | RPMI+10%FBS | 3.7 |
| 61 | RPMI+5%NCS | 4.0 | IMDM+1%BSA | 3.5 |
| 62 | RPMI+0.5%BSA | 4.0 | DMEM+1.5%Albumax | 3.5 |
| 63 | RPMI+No protein | 4.0 | DMEM+1%Albumax | 3.3 |
| 64 | RPMI+1%BSA | 3.6 | RPMI+1.5%BSA | 3.3 |
| 65 | RPMI+10%NCS | 3.6 | RPMI+5%FBS | 3.1 |
| 66 | RPMI+15%NCS | 3.5 | DMEM+No protein | 3.0 |
| 67 | RPMI+15%FBS | 3.5 | IMDM+0.5%Albumax | 3.0 |
| 68 | RPMI+1.5%BSA | 3.3 | RPMI+1%BSA | 2.5 |
| 69 | DMEM+15%NCS | 3.1 | IMDM+1.5%Albumax | 2.4 |
| 70 | DMEM+No protein | 3.0 | IMDM+1.5%BSA | 2.3 |
| 71 | RPMI+10%FBS | 2.2 | IMDM+No protein | 2.2 |
| 72 | RPMI+5%FBS | 1.5 | RPMI+No protein | 2.0 |
| 73 | RPMI+1.5%Albumax | 0.9 | RPMI+LLC-MK2+0.5%Albumax | 1.3 |
| 74 | RPMI+LLC-MK2+0.5%Albumax | 0.8 | RPMI+LLC-MK2+1.5%Albumax | 1.3 |
| 75 | RPMI+LLC-MK2+1%Albumax | 0.6 | RPMI+LLC-MK2+1%Albumax | 1.2 |
| 76 | RPMI+0.5%Albumax | 0.5 | RPMI+0.5%Albumax | 1.2 |
| 77 | RPMI+1%Albumax | 0.5 | RPMI+1.5%Albumax | 1.2 |
| 78 | RPMI+LLC-MK2+1.5%Albumax | 0.4 | RPMI+1%Albumax | 1.1 |

*The lower the rank, the better the system
