# Supplementary material for: Evaluation of in vitro culture systems for the maintenance of microfilariae and infective larvae of Loa loa
Source: Parasit Vectors. 2018 May 2;11:275. doi: 10.1186/s13071-018-2852-2 (PMC5930665; doi:10.1186/s13071-018-2852-2)
Supplement: Supplementary file 3 — Table S3. Moulting rate (%) of L. loa L3 in different in vitro culture systems. (DOCX 17 kb) [file 13071_2018_2852_MOESM3_ESM.docx]

**Additional file 3: Table S3.** Moulting rate (%) of *L. loa* L3 in different *in vitro* culture systems

| Serum/protein | Concentration (%) | DMEM | | IMDM | | RPMI | |
| --- | --- | --- | --- | --- | --- | --- | --- |
|  |  | LLC-MK2 | No feeder cells | LLC-MK2 | No feeder cells | LLC-MK2 | No feeder cells |
| No protein | 0 | 18.9±7.7 | 0 | 7.6±7.3 | 25.1±10 | 1.3±3.5 | 0 |
| Albumax |  |  |  |  |  |  |  |
|  | 0.5 | 29.6±13.6 | 0 | 16.9±9 | 6.1±9.5 | 0 | 0 |
|  | 1 | 31±12.6 | 0 | 4.8±11.7 | 3.1±6.2 | 0 | 0 |
|  | 1.5 | 22.3±18.2 | 0 | 6.7±16.3 | 7±9 | 0 | 0 |
| BSA | 0.5 | 59.2±23 | 3.3±2.9 | 29.7±21.2 | 5.6±13.6 | 0 | 0 |
|  | 1 | 64.1±29.4 | 0 | 17.8±17.3 | 0 | 0 | 0 |
|  | 1.5 | 58.6±10.7 | 0 | 35.9±26.9 | 0 | 0 | 0 |
| FBS | 5 | 29.9±16.4 | 14.6±13.7 | 12.3±13.8 | 9.2±13.1 | 3±5.1 | 0 |
|  | 10 | 22.8±8.1 | 26.2±7.1 | 4.1±6.6 | 8.2±13.8 | 3.4±6.7 | 0 |
|  | 15 | 13.4±7.5 | 18±17.9 | 1.7±4.1 | 5.8±6.6 | 0.8±2 | 0 |
| NCS | 5 | 34.7±17.5 | 30.1±6.3 | 15.8±9.6 | 19.9±31.2 | 0 | 0 |
|  | 10 | 28.9±9.6 | 21.9±17.3 | 18.4±7.5 | 15.1±11.8 | 0 | 0 |
|  | 15 | 27.8±12.5 | 13.6±14.3 | 15.8±13.8 | 10±13 | 0 | 0 |
