# Supplementary material for: Evaluation of in vitro culture systems for the maintenance of microfilariae and infective larvae of Loa loa
Source: Parasit Vectors. 2018 May 2;11:275. doi: 10.1186/s13071-018-2852-2 (PMC5930665; doi:10.1186/s13071-018-2852-2)
Supplement: Supplementary file 4 — Figure S1. Motility standardized residual histogram of the motility. (DOCX 41 kb) [file 13071_2018_2852_MOESM4_ESM.docx]

**Additional file 4: Figure S1.** Motility standardized residual histogram of the motility


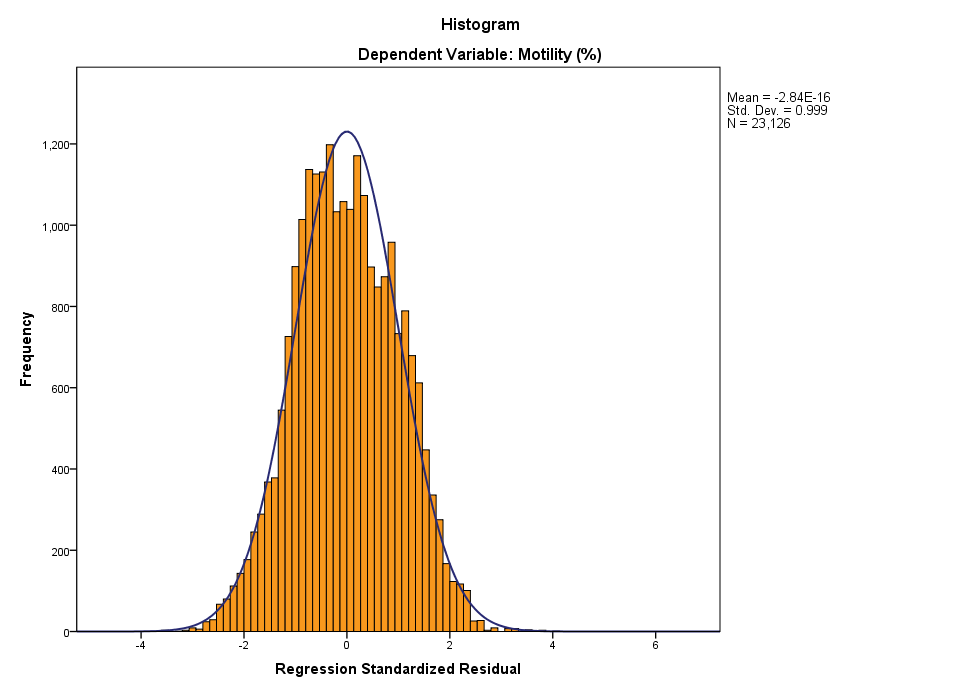


The histogram of the residuals (errors) in the model was used to check if they are normally distributed. Although not perfect, the frequency distribution of the residuals has a shape close to that of the normal Gauss curve, indicating evidence of normal distribution. P-P plot was used for further check.
