# Supplementary material for: Evaluation of in vitro culture systems for the maintenance of microfilariae and infective larvae of Loa loa
Source: Parasit Vectors. 2018 May 2;11:275. doi: 10.1186/s13071-018-2852-2 (PMC5930665; doi:10.1186/s13071-018-2852-2)
Supplement: Supplementary file 5 — Figure S2. Gaussian regression P-P plot of predicted motility. (DOCX 27 kb) [file 13071_2018_2852_MOESM5_ESM.docx]

**Additional file 5: Figure S2.** Gaussian regression P-P plot of predicted motility


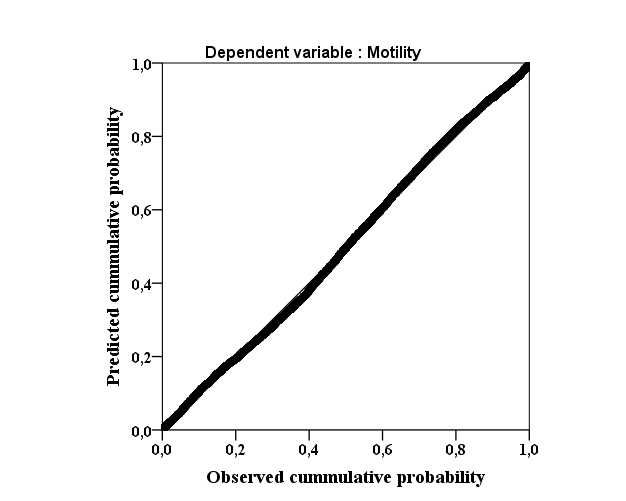


Additionally, the normal distribution was check using a P-P plot. Here, the expected and observed cumulative probabilities were closed suggesting that the assumption of normal distribution of the residual was far to be not violated.
