# Supplementary material for: Evaluation of in vitro culture systems for the maintenance of microfilariae and infective larvae of Loa loa
Source: Parasit Vectors. 2018 May 2;11:275. doi: 10.1186/s13071-018-2852-2 (PMC5930665; doi:10.1186/s13071-018-2852-2)
Supplement: Supplementary file 6 — Figure S3. Scatterplot of standardized residuals against standardized predicted values. (DOCX 29 kb) [file 13071_2018_2852_MOESM6_ESM.docx]

A**dditional file 6: Figure S3.** Scatterplot of standardized residuals against standardized predicted values


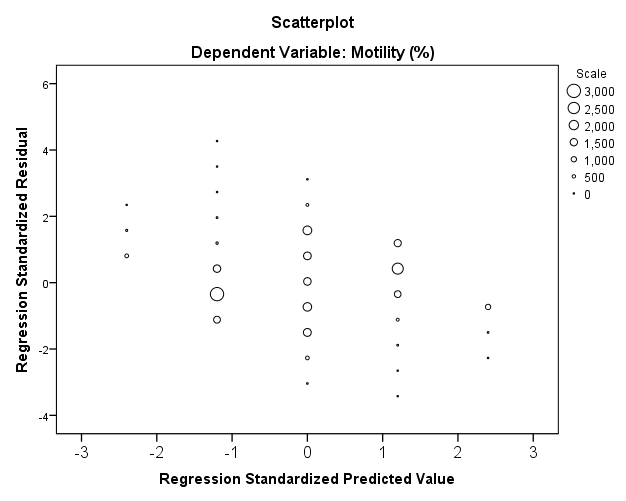


In this scatterplot of standardized residuals against standardized predicted values, the points are randomly spread out indicating that the assumption of homoscedasticity is likely to be safe
